# The Impact of the COVID-19 Pandemic on Esophageal and Gastric Cancer Surgery in Germany: A Four-Year Retrospective Single-Center Study of 287 Patients

**DOI:** 10.3390/jcm13061560

**Published:** 2024-03-08

**Authors:** Marius Ibach, Axel Winter, Philippa Seika, Paul Ritschl, Nadja Berndt, Eva Dobrindt, Jonas Raakow, Johann Pratschke, Christian Denecke, Max Magnus Maurer

**Affiliations:** 1 Chirurgische Klinik, Campus Charité Mitte/Campus Virchow-Klinikum, Charité Universitätsmedizin Berlin, 13353 Berlin, Germany; 2 Berlin Institute of Health at Charité—Universitätsmedizin Berlin, BIH Biomedical Innovation Academy, BIH Charité Clinician Scientist Program, Charitéplatz 1, 10117 Berlin, Germany

**Keywords:** COVID-19, oncology, surgery, gastrointestinal cancer, esophagus, stomach, perioperative outcomes, neoadjuvant therapy, impact

## Abstract

**Background:** Disruptions to surgical care for cancer patients during the COVID-19 pandemic remain an ongoing debate. This study assesses the effects of the COVID-19 pandemic on perioperative outcomes in a continuous series of surgically treated esophageal and gastric carcinoma patients at a large university hospital in Europe over 48 months. **Methods:** We conducted a retrospective single-center cohort study at a tertiary referral center. All patients who underwent oncologic esophageal or gastric resection between March 2018 and February 2022 were included in the analysis. The sample was split into a 24 months COVID-19 and an equivalent pre-COVID-19 control period. Outcome variables included caseload, in-hospital mortality, morbidity, treatment course, and disease stage at presentation. **Results:** Surgeons performed 287 operations, with around two-thirds (62%) of the cohort undergoing esophagectomy and one-third (38%) gastrectomy. The in-hospital mortality was 1% for the COVID-19 and the control periods. Patients did not present at a later disease stage nor did they wait longer for treatment. There was no decrease in caseload, and patients did not suffer from more perioperative complications during COVID-19. **Conclusions:** Esophageal and gastric carcinoma patients received safe and timely surgical care during the pandemic. Future pandemic protocols may streamline oncologic care towards tertiary referral centers.

## 1. Introduction

In March 2020, Germany prepared to face the rapid spread of the novel COVID-19 virus [1,2]. In response, authorities reallocated and diverted healthcare resources to cope with the consequences of an evolving pandemic: hospitals created new units and redeployed staff but also had to reduce surgical procedures to disburden intensive care facilities [3,4]. As a result, Germany maintained one of the lowest COVID-19 mortality rates internationally over the subsequent two pandemic years [5]. However, concerns have been raised that the emphasis on managing the COVID-19 pandemic may have negatively affected regular treatment regimens—particularly those of cancer patients. For patients requiring oncologic resection, therapy disruptions such as delayed treatment or decreased staffing have been shown to increase mortality [6,7].

Surgical treatment for upper gastrointestinal malignancy—including esophageal (EC), gastroesophageal junction (GEJ), and gastric cancer (GC)—necessitates highly complex procedures and requires swift management to prevent disease progression and death [8,9]. So far, the impact of the COVID-19 pandemic healthcare measurements on surgical care for these malignancies remains unclear. Prior evidence is scarce and conflicting, ranging from negligible effects to treatment delays and potential mortality increases in the early phase of the pandemic [10,11,12,13,14]. Given possible disruptions to care, we hypothesized that the perioperative morbidity and mortality in oncologic upper gastrointestinal surgery and the time to treatment after neoadjuvant therapy would have increased compared to pre-COVID-19 routine services.

In this study, we use data on perioperative outcomes of upper gastrointestinal surgery from a large European university hospital center to compare the two years following the introduction of pandemic policies in Germany with a two-year control period from our routine services before the pandemic outbreak. The results will assist the discussion within the medical and public debate about whether policy measures were appropriate—particularly concerning care for cancer patients.

## 2. Materials and Methods

This was a retrospective single-center cohort study to evaluate the impact of the COVID-19 pandemic on perioperative surgical outcomes after EG, GEJ, and GC resections compared to routine services at a tertiary university medical hospital in Germany. Data were obtained from the Surgical Department Charité Campus Mitte|Campus Virchow Klinikum database. The European data security protocol (DS-GVO) and the protocol for good clinical praxis of the Charité—Universitätsmedizin Berlin were followed.

**Figure 1:**
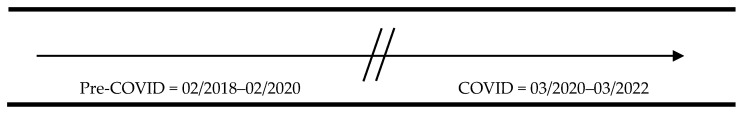
Study period.

Two corresponding periods were defined to assess the pandemic effect on surgical care: a 24-month COVID-19 period and an equivalent 24-month pre-COVID-19 control period (Figure 1). The COVID-19 period was defined from March 2020—when the German government initiated a pandemic response program—until February 2022. During these 24 months, authorities continually imposed moderate to complete lockdown restrictions, according to the Oxford COVID-19 stringency index [15]. We used this index in line with prior studies that have demonstrated a link between restriction severity on this index and surgical outcomes during the pandemic [14]. For controls, a pre-COVID-19 period was considered from March 2018 to February 2020, when routine surgical services occurred at our center.

Inclusion criteria comprised the following: (i) All patients undergoing oncologic resections for EC, GEJ, and GC at our center; (ii) Aged 18 years or older between February 2018 and March 2022. Several quantitative and qualitative perioperative outcomes were considered between the two time periods: differences in the number of surgeries per month and the monthly rate of change in surgeries were analyzed. Different qualitative measures, including perioperative mortality and morbidity, were investigated. Preoperative morbidity was estimated using the American Society of Anesthesiologists (ASA) physical status classification system [16]. We also compared the disease stage at presentation, differences in surgical approach, and the time passed between neoadjuvant therapy and surgery to investigate potential delays or changes in treatment protocols. The cancer stage at presentation was classified as early (UICC I/II) or advanced (UICC III/IV). Postoperative complications were categorized using the Clavien–Dindo Classification [17]. We also gauged postoperative morbidity by comparing the total hospital and intensive care unit stays between the two periods—since unobservable disruptions such as staffing shortages, equipment re-allocation, or disease progression may have translated into increased treatment duration.

Continuous variables are expressed as mean and standard deviation of the mean (SD) or as a count, including the percentage of the total (%). The cutoff value for statistical significance was set at alpha = 0.05. Continuous variables were tested for normality using the Kolmogorov–Smirnov and Shapiro–Wilk Test statistics. If variables were normally distributed, we used the *t*-test; otherwise, the Mann–Whitney U test for comparisons of the two groups was applied. Categorical data were compared using the Chi-square test statistic. Time series data were analyzed using the Dickey–Fuller test to check for non-stationarity of the absolute case numbers and monthly changes (first differences) in case numbers. A multivariate log-transformed linear regression model was used to test for potential effects on hospital and intensive care unit length of stay—as both variables were skewed to the right and non-normally distributed. All statistical analysis was performed using IBM SPSS Statistics software version 26 (SPSS Inc., Chicago, IL, USA) and R Statistical Software (version 4.3.1; R Foundation for Statistical Computing, Vienna, Austria).

## 3. Results

**Figure 2:**
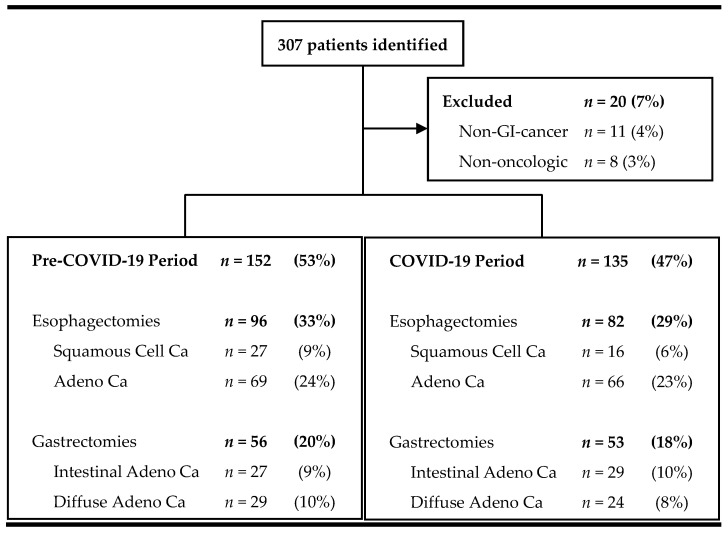
Flowchart of screened and included patients.

Baseline demographics—Between March 2018 and February 2022, 307 patients underwent esophageal or gastric resection at our tertiary university surgical department (Figure 2). Of those, 93% (*n* = 287) required surgery due to gastrointestinal malignancy and were included in the analysis.

The two periods did not exhibit major differences in the study population (Table 1): on average, patients were 63 years old (range: 27–89, *p* = 0.972), slightly overweight (BMI: 26 ± 5, *p* = 0.311), and more likely to be male (71%, *p* = 0.237). Most patients presented with reduced fitness and co-morbidities prior to surgery: 80% (*n* = 228) reported co-morbidities with a mean American Society of Anesthesiologists score (ASA) of 2.6 ± 0.6 across the entire study population. The most common secondary diagnoses included arterial hypertension (54%), diabetes mellitus (13%), and pulmonary disease (11%). Patients in the COVID-19 group displayed a lower percentage of stage one kidney failure (9% vs. 22%) and a higher percentage of patients with no kidney damage (44% vs. 35%) compared to the control group (*p* = 0.035).

**Figure 3:**
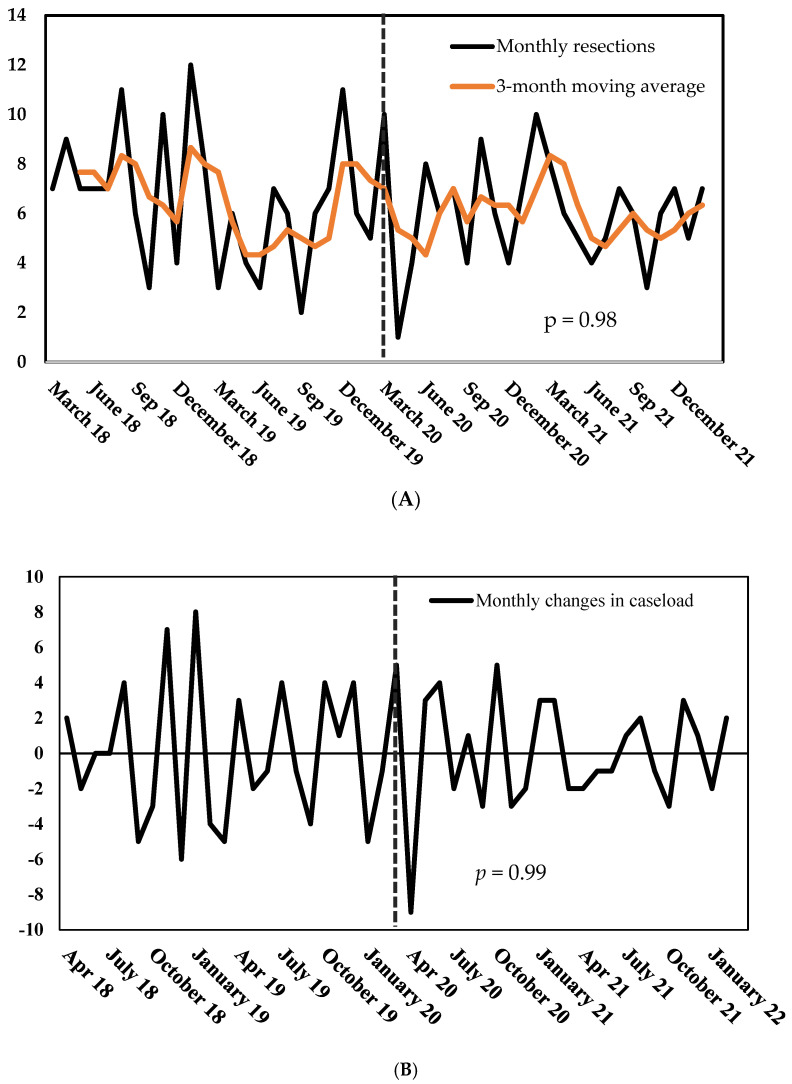
(**A**) Number of monthly gastric and esophageal resections, March 2018–March 2022. (**B**) Monthly changes in gastric and esophageal resections, March 2018–March 2022. *p* = probability of stationarity, Dickey–Fuller test.

Quantitative effects—Marking the pandemic’s beginning, the number of surgeries dropped by 90% from March to April 2020 (10 vs. 1, Figure 3A). However, the count of procedures swiftly recovered within three months. On average, the caseload changed by 67% per month before the pandemic and by 56% during the COVID period (*p* = 0.97, Figure 3B). Overall, the COVID-19 pandemic did not alter the caseload at our center (Figure 3A): surgeons performed more surgeries per month during the control period—however, this difference did not reach statistical significance (6.5 vs. 6.0, *p* = 0.47).

Qualitative effects: staging and neoadjuvant therapy—Patients in the pre-COVID-19 and COVID-19 periods did not vary significantly regarding histology and tumor stage. Of note, there was no significant increase in patients presenting with advanced compared to early tumor stages due to possible delays in diagnostics. Moreover, no difference in neoadjuvant therapy between the two time periods occurred (Table 2): most patients (91%, n = 261) received (Radio)-Chemotherapy as neoadjuvant therapy, with only a small percentage receiving none or with unknown status. Patients with gastric carcinoma received more cycles of neoadjuvant therapy during the COVID-19 period, but this difference did not reach statistical significance (4.3 vs. 3.7, *p* = 0.471).

Changes in Surgical Approach—All esophagectomies were conducted using the Ivor–Lewis procedure, and the surgical approaches included open, combined laparotomy/thoracotomy, laparoscopy, and robotic, with no significant difference between the groups (Table 3). The mean operating time decreased during COVID-19 compared to the control group (344 min vs. 415 min, *p* < 0.001). During the pandemic, surgeons at our center performed more robotic esophageal procedures (30% vs. 18%), but this difference did not reach statistical significance (*p* = 0.239).

For gastrectomies, performed as regular and transhiatal extended resections, the operating time did not differ between the COVID-19 and control groups. Most procedures were total gastrectomies (96% in the COVID-19 group and 88% in the control group, *p* = 0.163). The surgical approaches included open, hand-assisted laparoscopic surgery, conversion to open, and robotic, with no significant difference in distribution between the groups.

Perioperative outcomes—Patients waited two days longer for esophagectomy following neoadjuvant therapy during the COVID-19 period compared to the control period (47 vs. 45 days), but this difference did not reach statistical significance (*p* = 0.41, Table 4A). The distribution of Clavien–Dindo classifications showed no significant difference between the COVID-19 and control groups across all levels of morbidity and mortality following esophageal resection (*p* = 0.59). Similarly, there was no significant difference in median ICU (COVID-19 vs. control: 4 vs. 3 days, *p* = 0.65) or overall length of stay (14 vs. 17 days, *p* = 0.25). However, there was a significantly lower incidence of pneumonia in the COVID-19 group (16%) compared to the control group (26%, *p* = 0.01).

By comparison, there was no numerical difference in the time between chemotherapy and gastrectomy (COVID-19 vs. control: 42 vs. 42 days, *p* = 0.92, Table 4B). Similar to the esophagectomy group, the gastrectomy group showed no significant difference in the distribution of Clavien–Dindo classifications between the two periods (*p* = 0.39), indicating comparable morbidity and mortality. There was also no statistically significant difference in median days spent in the ICU (COVID-19 vs. control: 2 vs. 1, *p* = 0.07) and overall LOS (12 vs. 12 days, *p* = 0.51), as well as pneumonia rates (COVID-19 vs. control: 4% vs. 15% *p* = 0.07).

Across both groups, all patients underwent COVID-19 testing prior to surgery, and confirmed negative results were obtained for each patient. There was no case postponement due to an acute preoperative infection or a shortage of operating or intensive care unit capacities. In summary, postoperative outcomes, including mortality and morbidity as graded using the Clavien–Dindo classification, exhibited no difference between the two periods. However, there was a trend towards more patients suffering from pneumonia during routine services.

For 226 patients with all available data, we analyzed whether a significant relationship between the COVID-19 period and the length of stay and days in the intensive care unit would prove in a multivariable log-transformed linear regression model, as the outcome variables were right-skewed. General postoperative course predictors, including age, BMI, ASA, chronic kidney disease, and postoperative morbidity as represented using the Clavien–Dindo score, were controlled for in the analysis.

For the length of stay, a Clavien–Dindo score greater or equal to three was associated with a two-fold increase (105% = (e^0.72^ − 1) × 100; *p* < 0.001, Table 5). The ASA score also significantly affected the LOS: patients with an ASA of four spent three times more days in the hospital compared to those with a score of one (*p* < 0.01). Notably, the COVID-19 period was associated with a 17% decrease in the length of stay (*p* < 0.01). The surgery type, even when included as an interaction effect with the time variable, did not significantly affect the overall time spent in the hospital.

Using the same model, we found that an ASA score of four increased the days spent in the ICU five-fold (*p* < 0.01, Table 6). A complication rated as Clavien–Dindo grade three or greater was associated with a 30% increase in time in intensive care (*p* < 0.01). Unlike for the length of stay, neither the age nor the COVID-19 period were statistically significant, respectively. Notably, patients with gastrectomy stayed significantly shorter (46%) on the ICU compared to esophagectomy patients (*p* < 0.01). However, the interaction effect with the COVID-19 period was not significant, highlighting that this relationship was not specific to the pandemic.

## 4. Discussion

Overall, we found that the COVID-19 pandemic did not affect the amount or quality of upper gastrointestinal oncologic surgical care at a tertiary university surgical department in Germany. We found that patient numbers remained stable, and no significant changes in perioperative morbidity or mortality were to be observed. Of particular note, there was no delay in treatment: patients did not present in more advanced stages of the disease nor did they wait longer between neoadjuvant treatment and surgery as compared to routine services due to possible capacity restraints. This information is critical, as it demonstrates that a large tertiary referral hospital maintained care for cancer patients during the long-term upheaval caused by the pandemic. Prior research has demonstrated that the pandemic heightened anxiety among cancer patients, with many expressing fears of entering facilities treating COVID-19 patients [18,19]. However, given the results of our analysis, trust in hospital precaution measurements seems to have outweighed the fears induced by the pandemic as oncologic upper gastrointestinal surgery volume remained unaffected.

Interestingly, the total in-hospital stay was significantly shorter during the COVID-19 period compared to the pre-pandemic interval in the multivariable analysis, which might be interpreted as a more elaborate allocation of hospital resources at hand. Of note, this observation was not associated with a decrease in treatment quality in terms of postoperative complications or mortality. At the same time, ICU length of stay remained stable, underlining that this critical resource could be provided despite the high ICU demand during the COVID-19 pandemic. Moreover, a lower rate of postoperative pneumonia during the COVID-19 period could indicate that the use of personal protection equipment helped to shield patients from respiratory transmissions.

Prior research on upper gastrointestinal surgical care during COVID-19 exclusively focused on perioperative outcomes at the time of the outbreak in 2020, thereby generating conflicting evidence. In a single-center study from the United Kingdom, the in-hospital mortality for esophagectomies and gastrectomies was 4.2% and 0% from March to May 2020, respectively. The authors contribute these outcomes to logistics, communication, and excellent clinical care [10]. A research group from Boston (USA) reported comparable results: waiting time to surgery, pathological stage, perioperative morbidity, and mortality after esophagectomies remained stable at a large university hospital from March to June 2020 [11]. Other studies contrast with these findings: According to a survey conducted from March to May 2020 in twelve surgical centers in Italy, delays in surgery for esophageal cancer were reported in 37% of patients, while 14% had extended neoadjuvant therapy due to the pandemic. The authors attribute these delays to a shortage of anesthesiologists and the occupation of critical care beds by intubated COVID-19 patients [13]. In line with the Italian study, a single-center analysis from China showed that the waiting time for surgery and hospital stay length increased for patients treated for gastric cancer surgery from January to March 2020—indicating delays in treatment and increased perioperative morbidity or barriers to treatment [12]. In a multicenter survey conducted across 61 countries by the COVID Surge Collaborative between January and August 2020, total and medium lockdown restrictions on the Oxford stringency index were associated with a 15% and 6% non-operation rate for cancer surgery, respectively [14]. German authorities imposed comparable measures during the observational period of two years analyzed for the results presented here. However, we found no evidence of a decline in the operation rate or quality of care from a long-term perspective. This conflicting data indicates that the impact of the pandemic on cancer surgery depends on many factors, including the time frame under examination. During the first months of the upheaval, countries and healthcare systems adapted to the quickly emerging challenges, resulting in differences in perioperative outcomes. As the pandemic progressed—and the novelty of the circumstances decreased—hospitals and personnel may have successfully calibrated daily operations to meet the requirements of the pandemic. Our study suggests that, as a result of this calibration, healthcare teams were able to minimize the long-term effects of the pandemic on surgical oncologic care.

Another significant variable influencing perioperative outcomes during the pandemic may be the hospital size and the corresponding treatment capacity. Both the research from Boston and this study analyzed data from tertiary university hospitals. Contrastingly, the survey from Italy surveyed twelve different hospitals about a total of 65 patients over two months, indicating an average caseload of 2.7 per month—compared to 9 and 11 during the same period at our and the US center. Indeed, the authors of the Italian survey explain that only 50% of the centers in their analysis maintained routine caseloads. At the same time, the tertiary referral hospitals in their sample increased the number of esophagectomies to continue to provide overall care [11,13]. These findings indicate that hospital size was vital to continuous surgical cancer management and that tertiary referral centers can mitigate the stress placed during a pandemic, on healthcare systems.

From a long-term perspective, there are growing concerns that these results may only be applicable in the present. As of today, Germany benefits from a high intensive care unit bed per capita ratio [20]. This capacity allowed for the swift and strict separation of COVID-19 and non-COVID-19 patients. Nevertheless, this critical care capacity and, thus, the ability to withstand the pressures of a pandemic may decrease as a reduction in the total number of hospital beds to gain cost-effectiveness is currently discussed [21]. Even more important, hospitals can only utilize critical care beds when there is enough personnel to treat patients. There is growing evidence that hospital personnel might have exceeded their capabilities during the COVID-19 period. While many were motivated to serve the public in a global pandemic, we are registering an increasing number of critical care and nursing staff leaving the profession. Evidence suggests that the rate of nursing personnel quitting accelerated at the end of the pandemic, with many citing burnout and overwork as primary factors for their decision [22,23]. Thus, as healthcare capital in the form of hospital beds and the required labor force shrink, future healthcare systems, including the German system, may be less resilient to pandemic shocks.

Several limitations of this study have to be taken into account as this was a retrospective single-center analysis. On a national scale, access to care may have been limited for some patients: a country-wide analysis might show treatment delays or alterations in overall morbidity and mortality rates. Future research should also control for systemic factors like lockdown severity and hospital size when evaluating the pandemic’s impact on surgical care. The effects of the pandemic on direct surgical complications, such as anastomotic leakage rates, remain to be explored, highlighting an important area for future research to fully understand the nuances of surgical outcomes in the context of COVID-19. In addition, long-term effects may only become apparent once researchers can study survival rates over several years, highlighting the need for an extensive scope analysis.

In summary, this long-term study is the first to show that surgical treatment for gastric and esophageal cancer remained steady in a tertiary referral center throughout the pandemic in Germany. It addresses part of the ongoing debate about healthcare quality during these challenging times and underscores the vital role of large healthcare providers, the adaptability of modern hospital systems, and the necessity of a substantial healthcare workforce in mitigating a pandemic’s effects on cancer treatment. In the future, governments may develop emergency strategies focused on centralizing cancer care in tertiary referral centers with vast critical care capacities.

## 5. Conclusions

We found that the pandemic was not associated with increased mortality and morbidity in oncologic surgical patients with gastric and esophageal cancer at our center. We also found no delays in treatment or later disease presentations.

Our results suggest that it is possible to create a resilient surgical care center that continues to provide excellent care throughout a crisis such as the COVID-19 pandemic. Future studies should examine the relationship between hospital size and treatment maintenance during the pandemic to assist in creating robust healthcare systems.

## Figures and Tables

**Table 1:** Baseline demographics.

Characteristic	COVID-19	Control	*p*
N	135	152	
Age (years)	63 ± 11	63 ± 12	0.972
Male sex	102 (76%)	105 (70%)	0.237
BMI	26 ± 6	26 ± 4	0.311
Primary tumor			0.715
Esophagus, including AEG I, II	82 (61%)	96 (63%)	
Gastric, including AEG III	53 (39%)	56 (37%)	
ASA score, mean	2.6 ± 0.5	2.6 ± 0.6	0.414
ASA score categorization			0.582
1	1 (1%)	4 (3%)	
2	50 (37%)	59 (39%)	
3	87 (60%)	86 (57%)	
4	3 (2%)	2 (1%)	
Co-morbidities			
Yes	103 (76%)	125 (82%)	0.243
Cirrhosis	10 (7%)	15 (10%)	0.532
Pulmonary disease	16 (12%)	15 (10%)	0.704
Diabetes	22 (16%)	14 (9%)	0.077
CNS disorders	11 (8%)	12 (8%)	0.937
Coronary artery disease	14 (10%)	10 (7%)	0.289
Heart failure	9 (7%)	10 (7%)	0.976
Heart valve disease	6 (4%)	7 (5%)	0.948
Hypertension	73 (54%)	82 (54%)	0.983
Peripheral artery disease	4 (3%)	3 (2%)	0.588
Coagulation disorders	2 (2%)	3 (2%)	0.751
Autoimmune disease	1 (1%)	1 (1%)	0.933
Chronic kidney disease			0.035 *
0	59 (44%)	53 (35%)	
G1	12 (9%)	33 (22%)	
G2	52 (39%)	49 (32%)	
G3	9 (7%)	15 (10%)	
G4	1 (1%)	2 (1%)	
G5	1 (1%)	0	

* statistically significant at alpha = 0.05.

**Table 2:** Staging and Neoadjuvant Therapy.

Characteristic	COVID-19	Control	*p*
**Esophageal**	**N = 82**	**N = 96**	
Histology			0.220
Squamous cell carcinoma	16 (20%)	27 (28%)	
Adenocarcinoma	66 (80%)	69 (72%)	
Tumor stage			0.541
Early	46 (56%)	59 (62%)	
Advanced	36 (44%)	37 (39%)	
Neoadjuvant Therapy			0.99
(Radio)Chemotherapy	75 (92%)	87 (91%)	
None	4 (5%)	9 (9%)	
Unknown	3 (3%)	-	
Cycles	3.6 ± 1.6	3.7 ± 1.7	0.590
**Gastric**	**N = 53**	**N = 56**	
Histology			0.370
Diffuse Adenocarcinoma	24 (45%)	29 (52%)	
Intestinal Adenocarcinoma	29 (55%)	27 (48%)	
Tumor stage			0.391
Early	19 (36%)	15 (27%)	
Advanced	34 (64%)	40 (71%)	
Unavailable	-	1 (2%)	
Neoadjuvant Therapy			0.92
(Radio)Chemotherapy	51 (96%)	48 (86%)	
None	-	1 (2%)	
Unknown	2 (4%)	-	
Cycles	4.3 ± 2.2	3.7 ± 1.8	0.471

**Table 3:** Surgical Approach.

Characteristic	COVID-19	Control	*p*
**Esophagectomy**	**N = 81**	**N = 96**	
Operating time (min)	344 ± 72	415 ± 73	<0.001 *
Procedure			
Ivor–Lewis Esophagectomy	81 (100%)	96 (100%)	
Approach			0.239
Open	2 (2%)	1 (1%)	
Combined Laparotomy/Thoracoscopy	17 (18%)	14 (17%)	
Laparoscopy	48 (50%)	52 (63%)	
Robotic	29 (30%)	15 (18%)	
**Gastrectomy**			
Operating time	302 ± 100	316 ± 88	0.253
Procedure			0.163
Total	51 (96%)	49 (88%)	
Subtotal	2 (4%)	7 (13%)	
Approach			0.238
Open	23 (41%)	31 (59%)	
Hand-assisted laparoscopic surgery	28 (50%)	17 (32%)	
Conversion to open	2 (4%)	3 (6%)	
Robotic	3 (5%)	2 (4%)	

* statistically significant at alpha = 0.05.

**Table 4:** (**A**) Perioperative outcomes: Esophagectomy. Mean ± SD, count (percentage), or median (range). (**B**) Perioperative outcomes: Gastrectomy. Mean ± SD, count (percentage), or median (range).

**(A)**			
**Characteristic**	**COVID-19**	**Control**	***p***
	N = 98	N = 82	
Procedural			
Days between neoadjuvant therapy and surgery	47 ± 18	45 ± 19	0.41
Morbidity			
Complications			
Clavien–Dindo			0.59
I and II	15 (15%)	8 (10%)	
III	29 (20%)	26 (32%)	
IV	19 (19%)	18 (22%)	
V	3 (3%)	1 (1%)	
Clavien–Dindo ≥ 3	51 (52%)	45 (55%)	0.82
ICU (days)	4 (1–55)	3 (1–97)	0.65
Length of stay (days)	14 (8–110)	17 (10–107)	0.25
Pneumonia	13 (16%)	25 (26%)	0.01 *
Mortality			
Death within primary stay	3 (3%)	2 (1%)	1.00
**(B)**			
**Characteristic**	**COVID-19**	**Control**	***p***
	N = 50	N = 55	
Procedural			
Days between neoadjuvant therapy and surgery	42 ± 15	42 ± 15	0.92
Morbidity			
Complications			
Clavien–Dindo			0.39
I and II	4 (8%)	9 (16%)	
III	5 (10%)	4 (7%)	
IV	5 (10%)	4 (7%)	
V	0 (0%)	0 (0%)	
Clavien–Dindo ≥ 3	10 (20%)	8 (15%)	0.63
ICU (days)	2 (1–13)	1 (0–7)	0.07
Length of stay (days)	12 (7–21)	12 (8–60)	0.51
Pneumonia	3 (4%)	8 (15%)	0.07
Mortality			
Death within primary stay	0 (0%)	0 (0%)	1.00

* significant at alpha = 0.05.

**Table 5:** Log-Transformed Linear Regression Model. Length of stay (LOS) in days, *n* = 226.

Variable	Coefficient	Std. Error	t-Value	*p*-Value
Intercept	2.13	0.347	6.15	<0.01 *
COVID Period	−0.19	0.07	−2.99	<0.01 *
Age (y)	0.01	0.003	1.99	0.048 *
BMI	0.003	0.006	0.45	0.65
ASA 2	0.20	0.25	0.80	0.422
ASA 3	0.28	0.25	1.15	0.25
ASA 4	1.20	0.36	3.32	<0.01 *
CKD ^¥^ ≥ 3	0.114	0.11	1.02	0.31
Clavien–Dindo ≥ 3	0.72	0.07	10.81	<0.001 *
Gastrectomy	−0.01	0.08	−0.02	0.986

* statistically significant at alpha = 0.05. Effect size percentages can be estimated using the formula y = (e^β1^ − 1) × 100, where β1 is the coefficient for each variable; ^¥^ CKD = chronic kidney disease.

**Table 6:** Log-Transformed Linear Regression Model. Intensive care unit (ICU) in days, *n* = 226.

Variable	Coefficient	Std. Error	t-Value	*p*-Value
Intercept	−0.03	0.53	−0.03	0.98
COVID Period	0.11	0.10	1.09	0.28
Age (y)	0.01	0.01	1.58	0.12
BMI	0.002	0.01	0.25	0.80
ASA 2	0.30	0.38	0.80	0.43
ASA 3	0.61	0.38	1.62	0.11
ASA 4	1.95	0.55	3.53	<0.01 *
CKD ^¥^ > 3	0.119	0.17	0.70	0.49
Clavien–Dindo ≥ 3	0.27	0.13	2.08	<0.04 *
Gastrectomy	−0.62	0.13	−4.76	<0.01 *

* statistically significant at alpha = 0.05. Effect size percentages can be estimated using the formula y = (e^β1^ − 1) × 100, where β1 is the coefficient for each variable; ^¥^ CKD = chronic kidney disease.

## Data Availability

Retrospective data was collected from the Charité surgical database (not publicly available).
